# In vitro anti-proliferative activity of selected nutraceutical compounds in human cancer cell lines

**DOI:** 10.1186/s13104-020-05435-1

**Published:** 2021-01-07

**Authors:** Endalkachew Nibret, Sonja Krstin, Michael Wink

**Affiliations:** 1 Department of Biology, Science College, Bahir Dar University, P.O.Box 79, Bahir Dar, Ethiopia; 2 Biotechnology Research Institute, Bahir Dar University, P.O.Box 79, Bahir Dar, Ethiopia; 3 Institute of Pharmacy and Molecular Biotechnology, Heidelberg University, Im Neuenheimer Feld 364, 69120 Heidelberg, Germany

**Keywords:** Colorectal cancer, Lymphoblastic cancer, Nutraceuticals

## Abstract

**Objective:**

We investigated the anti-proliferative or cytotoxic activities of five nutraceutical compounds: allyl isothiocyanate, β-carotene, caffeine, capsaicin, and lupanine that we consume respectively, for example, from mustard seeds, carrot, coffee, pepper, and lupin seeds against cancer cell lines (human colon: HCT 116 p53 wild type, HCT 116 p53−/− and lymphoblastic: CEM/CCRF, CEM/ADR5000).

**Result:**

Out of the five compounds tested in vitro, capsaicin and β-carotene were more cytotoxic than the other three compounds against the four cancer cell lines. The most potent nutraceutical compound was capsaicin and it exerted its highest cytotoxicity against HCT 116 p53−/− with IC_50_ value of 19.67 ± 0.06 µM. It is worth considering capsaicin for further development of anticancer drug against both colon and leukemia cancer types.

## Introduction

Cancer is the second leading cause of death globally. In 2018 alone, an estimated 9.6 million deaths occurred due to cancer. Approximately 70% of these deaths occurred in low-and middle-income countries [1].

Some nutraceuticals that are consumed as food or part of food are known to provide health benefit in prevention and control of various infectious- and non-infectious diseases (e.g. cancer). To date, some nutraceuticals have been approved for clinical use [2, 3]. Still now, the search for new chemical entities having health benefit from food items is being intensively investigated. Quite recently, a few promising nutraceuticals entered clinical trials [4–6]. In addition, nutraceuticals could also exert negative effects in the consumer, which are often neglected in studies.

In the present study, we evaluated five nutraceuticals (allyl isothiocyanate, β-carotene, caffeine, capsaicin, lupanine) and the known anti-cancer drug, vinblastine, against four different types of cancer cell lines. Allyl isothiocyanate is one of the components of mustard paste prepared from mustard seeds and horseradish and is known for its pungency. It is derived from the glucosinolate sinigrin, which is found in some *Brassica* vegetables including cabbage, brussels sprouts, kale and cauliflower [7]. β-Carotene is one of the major carotenoids with antioxidant properties in our diet and its major sources of dietary include green leafy vegetables as well as orange and yellow fruits and vegetables [8]. Caffeine is an alkaloid, which is known for its mild stimulant and is traditionally consumed from sources like coffee, teas, cola, and chocolate [9]. Capsaicin is the active major alkaloid of hot chili and is responsible for pungent feeling of peppers in the genus *Capsicum*. Peppers are mainly consumed as food additives because of their unique pungency, aroma, and color [10]. Lupanine is one of the major quinolizidine alkaloids responsible for bitterness of lupins in the genus *Lupinus* [11, 12]. Lupin seeds are used in breadmaking, biscuits, pasta products, and a variety of other food products [13]. The food items containing the five nuraceuticals described in the present study are widely consumed in Ethiopia and elsewhere in other parts of the world. And therefore they were selected and evaluated against the human colon and leukaemia cancer cell lines. The colonrectal cancer (1.8 million cases) stands third in terms of affecting human health worldwide [1]. As our ongoing effort to search for active compounds, we also evaluated the nutraceuticals against the leukaemia cancer cell lines.

## Main text

### Methods

#### Chemicals and cell lines

Allyl isothiocyanate, beta-carotene, caffeine, capsaicin, vinblastine sulfate, fetal bovine serum (FBS), dimethyl sulfoxide (DMSO), and 3-(4,5-dimethylthiazol- 2-yl)-2,5-diphenyl-tetrazolium bromide (MTT) were purchased from Sigma-Aldrich GmbH, Steinheim, Germany. Lupanine was obtained from our laboratory (MW laboratory) which was extracted from *Lupinus* species previously [12]. RPMI-1640, DMEM, penicillin–streptomycin, trypsin–EDTA, and L-glutamine were purchased from Gibco, Karlsruhe, Germany.

Two adherent cancer cells (HCT 116, wild type colon cancer cells and HCT 116 p53 − / − , p53 knocked out colon cancer cells) and two other cancer cells in suspension (CEM/CCRF, T-lymphoblastic leukemia and CEM/ADR5000, T-lymphoblastic leukemia over-expressing P-gp) have been routinely cultured in our laboratory for various research works. HCT 116 p 53 − / − , p53 cells were obtained from Prof. Stefan Wölfl group, Institute of Pharmacy and Molecular Biotechnology, Heidelberg University. CEM/ADR5000 and CEM/CCRF cell lines were originally obtained from Professor T. Efferth, Department of Pharmaceutical Biology, University of Mainz, Germany, and maintained in Prof. M. Wink’s laboratory, Institute of Pharmacy and Molecular Biotechnology, Heidelberg University.

#### Cell culture

The human cells, CEM/CCRF cells and P-gp over-expressing CEM/ADR 5000, were cultured in RPMI 1640 medium supplemented with 10% (v/v) FBS, 2 mM L-glutamine, and 100 U/ml penicillin, and 100 g/ml streptomycin.

HCT 116 cell lines were maintained in DMEM supplemented with 10% (v/v) FBS, 2 mM L-glutamine, 100 U/ml penicillin, and 100 g/ml streptomycin. HCT 116 cells were detached from the culture vessel by adding trypsin–EDTA for 5 min. Cells were cultivated at 37 °C, 5% CO_2_, and 95% humidity.

#### Cytotoxicity assays

Cells growing in logarithmic growth phase were counted using haemocytometer and then the number of cells to be seeded to 96-well plates were adjusted at 5 × 10^3^/ml. HCT 116 and HCT 116 p53 − / − were then incubated for 24 h at 37 °C, 5% CO_2_. Stock solutions of test compounds were prepared using DMSO. Ten different concentrations of the test compounds which were diluted in two-fold fashion in the medium were then added to the wells and incubated further for 48 h [14] and the viability of the cells was determined using the MTT assay [15]. MTT solution (0.5 mg/ml) was added to wells and the optimum incubation of the HCT 116 cells with MTT was 3 h. After removing the liquid from the wells, 100 µl of DMSO was added to dissolve the formazan crystals produced by the cells. The 96-well plates were shaken for 10 min and the optical density was measured at 570 nm using a Tecan microplate reader (Crailsheim, Germany). Unlike HCT 116 cells, CEM/CCRF and CEM/ADR 5000 cells were seeded to the 96-wells at 5 × 10^4^/ml and then treated with test compounds for 48 h. The optimum incubation time of these cells with MTT was 4 h. Each test compound was evaluated in triplicate and repeated at least two times. The 50% inhibitory concentration (IC_50_) of each test compound against the cancer cells was determined using SigmaPlot 11.0 software (Systat Software Inc., San Jose, CA, USA).

### Results and discussion

There is ample evidence that mutations in the p53 tumor-suppressor gene are prevalent in human cancers [16] and cells bearing these mutations are rendered to be relatively resistant to drugs. Our results corroborate this feature in which we showed that p53 knockout (p53 − / −) HCT 116 cells were more resistant to allyl isothiocyanate (AITC) than their counterpart p53 wild type (*p*53 + / +) (Table 1). Reactivating p53 mutant to wild type tumor-suppressive function was possible in cancer cells *(*e.g. SK-BR-3 cells) using other types of isothiocyanates (e.g. phenethyl isothiocyanate PEITC) both in vitro and in vivo. It was shown that PEITC induces apoptosis by restoring p53 wild type conformation in mutant cells, revealing a new mechanism of action for a dietary-related compound against cancer cells [16]. However, Pappa et al. [17] suggested that PEITC induces apoptosis in both types of HCT 116 cells in a p53-independent manner. In rat model, AITC-rich mustard seed powder at 71.5 mg/kg oral dose appeared to be more robust than that of pure AITC and inhibited bladder cancer growth by 34.5% and blocked muscle invasion by 100%, suggesting the anticancer properties of nutraceuticals [18].

**Table 1:** Anti-proliferative activity of some nutraceutical compounds against human colon cancer and lymphoblastic leukemia cell lines

Compound		IC_50_ (µM)		
	HCT116	HCT116 p53 − / −	CEM/CCRF	CEM/ADR5000
Allyl isothiocyanate	162.92 ± 1.68	> 500	155.76 ± 0.62	194.99 ± 3.87
β-Carotene	139.53 ± 5.72	34.94 ± 0.47	34.46 ± 1.2	220.58 ± 12.25
Caffeine	> 500	332.07 ± 14.05	444.71 ± 8.29	> 500
Capsaicin	22.21 ± 0.49	19.67 ± 0.06	64.56 ± 2.40	122.98 ± 0.10
Lupanine	> 500	> 500	> 500	> 500
Vinblastine sulfate	0.81	< 0.015	0.14	6.37

In our present study, CEM/ADR5000 cells were also more resistant to AITC than CEM/CCRF cells (194.99 ± 3.87 µM versus 155.76 ± 0.62 µM). CEM/ADR5000 cells are known to overexpress transmembrane efflux pumps such as ATP-binding cassette (ABC) transporters (e.g. p-gp, BCRP and ABCB5). Although multidrug resistance (MDR) is a multi-factorial process, the current finding, among others, might be ascribed to P-gp as we know from the very beginning that the cultured CEM/ADR5000 cells do overexpress P-gp [19]. Generally, the isothiocyanates are capable of forming covalent bonds with amino groups of amino acid residues (e.g., lysine, arginine) of proteins and also with primary amino groups of DNA bases that would result in protein and DNA alkylation. This alkylation property of isothiocyanates in part explains their mechanism of action for their superb biological activity [20].

In our study, the IC_50_ ratio ( IC_50_ against (p53 − / −) HCT 116 to IC_50_ against (p53 + / +) HCT 116)) clearly show that p53 knockout HCT116 cells were about four times more sensitive to β-carotene than p53 wild type HCT 116 cells, suggesting the use of this compound against colon cancers cells like HCT 116 bearing p53 mutations. A number of studies have shown anti-proliferative activity of β-carotene against different types of cancer. A recent study by Kim et al. [21] showed that β-carotene elevated histone H3 and H4 acetylation upon the treatment of HCT 116 colon cancer stem cells, which is suggestive of an epigenetic modification for anti-proliferative activity of β-carotene.

In the present study, the leukemia cell lines CEM/CCRF cells were more sensitive to β-carotene than their corresponding drug resistant CEM/ADR5000 cells. We have shown previously that carotenoids (e.g., β-carotene) as competitive inhibitors of ABC-transporter with the ability of reversing MDR in P-gp expressing cells, which is suggestive of their utilization as adjuvants in chemotherapy [22].

Caffeine is the least active nutraceuticals next to lupanine against the four cancer types tested (Table1). Higher concentrations of caffeine were required to have anti-proliferative activity against P53 knockout HCT 116 colon cancer cells and CEM/CCRF leukemia cells. IC_50_ values of caffeine against p53 wild type HC116 and CEM/ADR5000 were even higher than the highest concentration tested (500 µM). Kaplanek et al. [23] evaluated caffeine up to 25 µM for 72 h and found IC_50_ to be higher than 25 µM against seven cancer cell lines including the four cancer cell types what we investigated currently. Nevertheless, these authors demonstrated a significant suppressing effect on proliferation by increasing the doses of caffeine (the maximum inhibition being achieved at 5000 µM). Quite recently in our laboratory, we demonstrated caffeine as having protective effect against acute oxidative stress and extending the lifespan of *Caenorhabditis elegans* worms [24].

**Fig. 1:**
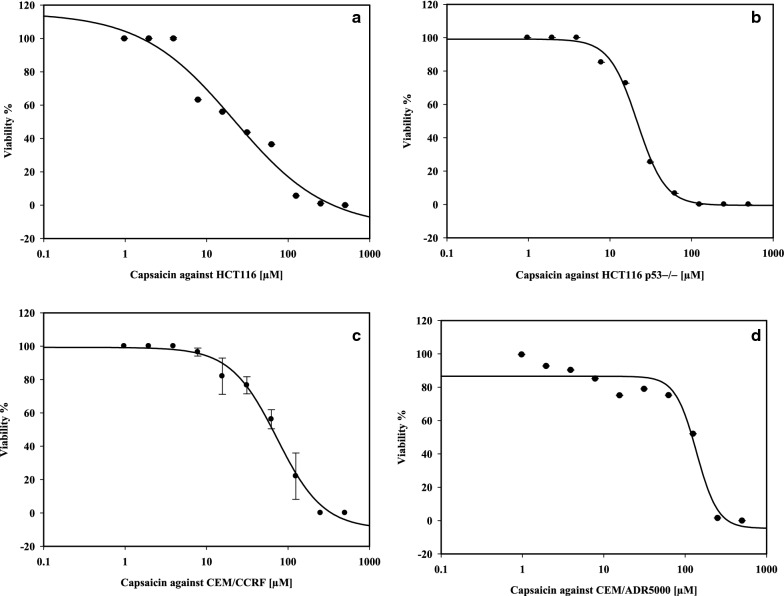
The anti-proliferative activity of capsaicin against **a** HCT 116 p53 wild type **b** HCT 116 P53−/− **c** CEM/CCRF **d** CEM/ADR5000 cancer lines

Capsaicin is the most active nutraceutical compound against the four types of cancer cell lines (Fig. 1) (Additional file 1: Additional graphs). Its highest cytotoxicity activity was exerted against p53 knockout HCT116 cell line (IC50: 19.67 µM), indicating these types of cells to be more sensitive to capsaicin than their corresponding p53 wild type HCT116 cells. A study by Senawong et al. [25] demonstrated the anti-proliferative activity of capsaicin without cell-cycle arrest property against HCT 116 cells. Furthermore, it was shown that pepper seed extract, at a concentration of 500 µg/ml, exhibited greater anti-proliferative activity (96.8%) and increased apoptotic cell population by 2.2-fold in HCT 116 cell line [26]. Like AITC discussed above, capsaicin was shown to restore wild type p53 activities by degrading mutant p53 protein in other human cancer lines U373 (glioblastoma) and SKBR3 (breast cancer) carrying p53 mutation, rendering cancer cells to be more susceptible to cancer drugs [27]. However, caution should be taken in using capsaicin at low concentration for the treatment of colon cancer as it was confirmed that it enhanced both migratory and invasive capability of HCT 116 cells both in vitro and in vivo [28, 29].

CEM/CCRF cells were about two times more sensitive to capsaicin than their corresponding CEM/ADR5000 cells. In this study, it was worth noting that, compared to other nutraceutical compounds tested against drug resistant CEM/ADR5000, capsaicin exerted its cytotoxicity with the lowest IC_50_ value of 122.98 µM. Cetintas et al. [30] also found a comparable finding (IC_50_ = 80 µM using WST-1 assay) in which they showed the anti-proliferative activity and apoptosis induction property of capsaicin in CEM/CCRF cells evidenced by an increase caspase-3 activity and a decrease in Bcl-2 gene mRNA and protein expression. Like β-carotene, we showed previously in our laboratory the ability of capsaicin reversing multidrug resistance in P-gp overexpressing Caco-2 and CEM/ADR5000 cell lines [14].

Lupanine is the least active nutraceutical compound tested against the four cancer cell lines and its IC_50_ value could not be calculated as it was greater than the maximum concentration, 500 µM, tested. No other study, other than ours, reported the effect of this compound against cancer cell lines. However, lupanine and other quinolizidine alkaloids (QA) are neurotoxins, affecting acetylcholine receptors and ion channels. And it is therefore only those lupin seeds with low concentrations of QA (below 200 µg/g seeds) are allowed for human consumption [31].

In conclusion, capsaicin is the most potent anti-proliferative nutraceutical followed by beta-carotene. Our results and other previous findings showed that the high concentration of capsaicin as having potent anti-proliferative activity without inducing migratory and invasive property of colon cancer cells both in vitro and in vivo.

### Limitation

The cytotoxicity assay was assessed using only MTT, which is not sensitive to minor changes. Moreover, additional data should have been presented using microscope which show the changes in cells to explore mechanisms by which nutraceutical compounds caused cell death.

## Supplementary Information

**Additional file 1.** Additional graphs.

## Data Availability

The readers may contact the corresponding author to access the raw data used for the calculation of IC50 values using Sigmaplot11.

## Abbreviations

AITC: Allyl isothiocyanate
DMSO: Dimethyl sulfoxide
IC_50_: 50% Inhibitory concentration of test compound
PEITC: Phenethyl isothiocyanate
MTT: 3-(4,5-Dimethylthiazol- 2-yl)-2,5-diphenyl-tetrazolium bromide
