# Supplementary material for: In vitro anti-proliferative activity of selected nutraceutical compounds in human cancer cell lines
Source: BMC Res Notes. 2021 Jan 7;14:18. doi: 10.1186/s13104-020-05435-1 (PMC7791773; doi:10.1186/s13104-020-05435-1)
Supplement: Supplementary file 1 — Additional file 1. Additional graphs. [file 13104_2020_5435_MOESM1_ESM.doc]

**Additional graphs showing the effect of nutraceuticals on cancer cell lines**
